# Immunomodulatory role of bitter melon extract in inhibition of head and neck squamous cell carcinoma growth

**DOI:** 10.18632/oncotarget.8898

**Published:** 2016-04-21

**Authors:** Sourav Bhattacharya, Naoshad Muhammad, Robert Steele, Guangyong Peng, Ratna B. Ray

**Affiliations:** ^1^ Department of Pathology, Saint Louis University, Saint Louis, Missouri, USA; ^2^ Department of Internal Medicine, Saint Louis University, Saint Louis, Missouri, USA

**Keywords:** bitter melon, TME, HNSCC

## Abstract

Head and neck squamous cell carcinoma (HNSCC) is the sixth most common cancer and leading cause of cancer related mortality worldwide. Despite the advancement in treatment procedures the overall survival rate of patients has not considerably enhanced in the past few decades. Therefore, new strategies to achieve a favorable response for the improvement in the prognosis of HNSCC are urgently needed. In this study, we examined the role of bitter melon extract (BME) in HNSCC tumor microenvironment. Mouse head and neck cancer (SCCVII) cells were subcutaneously injected into the flanks of syngeneic mice. We observed that oral gavage of BME significantly inhibits the tumor growth in mice as compared to control group. Further study suggested that BME inhibits cell proliferation as evident from low expression of proliferating cell nuclear antigen (PCNA) and c-Myc in the tumors of BME fed mice as compared to that of control group. We next investigated the role of BME as an immunomodulator in HNSCC model. Forkhead box protein P3^+^ (FoxP3^+^) T cells suppress tumor immunity. Our data suggested that BME treatment decreases the infiltrating regulatory T (Treg) cells by inhibiting FoxP3^+^ populations in the tumors and in spleens. Additionally, BME treatment reduces Th17 cell population in the tumor. However, BME treatment did not alter Th1 and Th2 cell populations. Together, our findings offer a new insight into how bitter melon extract inhibits head and neck tumor growth by modulating cell proliferation and Treg populations, with implications for how to control tumor-infiltrating lymphocytes and tumor progression.

## INTRODUCTION

Head and neck squamous cell carcinoma (HNSCC) is the sixth most prevalent cancer and leading cause of cancer related mortality worldwide. In the United States, 50,000 new cases are diagnosed, and nearly 10,000 deaths are attributed to this disease annually. However, the overall 5-year survival rate remains unchanged (50-55%) over several decades. Although improvements in surgical techniques, chemotherapy and radiation delivery, and supportive care have improved quality of life for patients with HNSCC, the regional and distant recurrence remains common and is almost always fatal. Thus, there is a great need to develop more effective therapies for patients with HNSCC to increase cure rates and reduce morbidity.

Natural products play a leading role in the discovery and the development of various drugs for the treatment of human diseases including cancer. Several epidemiological and preclinical studies have shown that diet rich in fruit and vegetable reduces the risk of several types of cancer [1, 2]. Bitter melon (*Momordica charantia*) is extensively cultivated in Asia, Africa, and South America, and widely used in folk medicines to treat diabetes [1]. We have previously reported that BME inhibits HNSCC cell proliferation through modulation in c-Met signaling in *in vitro* studies as well as in xenograft model of HNSCC [3].

Several evidences support that the suppressive tumor microenvironment, where other cells (especially immune cells) cross-talk with tumor cells, is an obstacle for effective anti-tumor immunity and successful tumor immunotherapy [4, 5]. Regulatory T (Treg) cells are recruited into neoplastic tissues by cytokines, most notably CCL2 and TGF-β; and their abundance correlates with poor outcome in HNSCC [6]. Therefore, Tregs are a key component forming the immune-suppressive microenvironment, which are corrupted to dampen anti-tumor immunity [7]. Current immunotherapies for cancer face the challenges of severe side effects [8]. Naturally occurring anti-inflammatory or immunomodulatory plant extracts contribute to anticancer effect by alteration of immune signaling pathways [9]. However, the role of BME as an immunomodulator in HNSCC has not been studied. In this study, we demonstrated that BME treatment in a syngeneic mouse model of head and neck cancer not only inhibits tumor cell proliferation but also modulated Treg cell population within the tumor suppressive microenvironment. To our knowledge, this is the first report demonstrating BME exerts immunomodulatory effect in regressing HNSCC tumor growth in a preclinical model.

## RESULTS

### Treatment of bitter melon extract inhibits tumor progression

**Figure 1:**
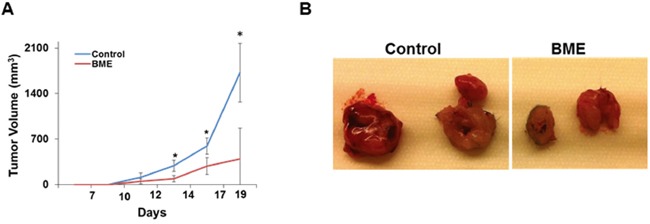
Oral administration of BME in syngeneic mice inhibits tumor growth **A.** SCCVII cells were implanted subcutaneously into the flank of C3H mice. Tumor bearing mice were randomized into two groups, and water (control) or BME was gavaged orally for ~3 weeks (5 days/week). Volume of tumor growth was monitored as indicated time points and presented as a mean. Small bar indicates standard error (*, p<0.05). **B.** Representative tumors dissected from control and BME-fed mice.

We have previously reported that BME feeding regress tumor growth in Cal27 xenograft model [3], although the effect of BME on HNSCC in presence of intact immune system remains unknown. Here, we examined the effect of BME in suppression of the tumor growth in the syngeneic mouse model of head and neck cancer. Mouse HNSCC (SCCVII) cells were implanted into the flanks of mice. Mice were divided into two groups. Mice received 100 μl water (control group) or 100 μl BME by oral gavage (experimental group) 5 days/week for the entire experimental timeframe as described previously [3, 10]. The dose of BME is determined based on our previous experiences [3, 10]. Tumor volume was measured at indicated time points and our results showed that BME treatment reduces the tumor growth as compared to control group (Figure 1, panel A). Representative images of the tumors are shown in Figure 1 (panel B). Our results clearly suggested that BME significantly inhibited HNSCC tumor growth. We further examined *in vitro* efficacy of SCCVII cells following treatment with BME using different doses, and cell viability was determined. A dose dependent effect was observed (Supplementary Figure S1).

### Bitter melon modulates cell proliferation

**Figure 2:**
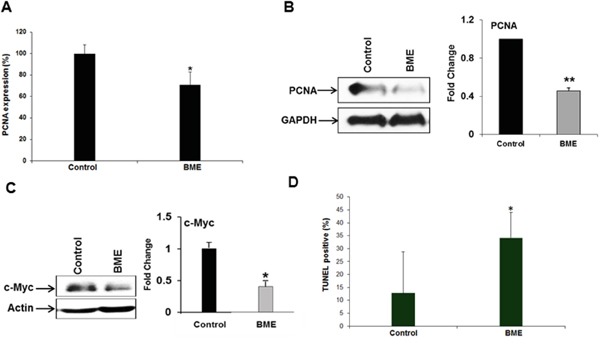
BME treatment inhibits cell proliferation **A.** Quantitation of immunohistochemical staining for the PCNA in tumor sections of control and BME fed mice. **B** and **C.** Western blot analyses of PCNA and c-Myc expression in tumor tissues isolated from both control and BME-fed mice. The blot was reprobed with an antibody to GAPDH or actin for comparison of protein load. Densitometry scanning for quantitation is shown on the right. (*, p<0.05; **, p<0.001). **D.** Measurement of apoptosis in the tumors was examined by TUNEL assay and quantitation of % TUNEL positive nuclei from control and BME-fed mice is shown.

Since we observed smaller tumor volumes in BME-fed mice, we examined the status of PCNA in tumors to study the mechanism. PCNA is required for cell growth and cell cycle progression in mammalian cells. The tissue samples from control and experimental mice were analyzed by PCNA immunostaining. Quantitative microscopic examination of PCNA-stained sections showed that PCNA-positive cells in BME-fed group were ~70% as compared with control group (Figure 2, panel A). Western blot analysis of PCNA was further confirmed that PCNA expression was indeed inhibited in BME-fed mice as compared with control mice (Figure 2, panel B), suggesting impairment of cell proliferation. c-Myc expression was inhibited by BME in a HNSCC xenograft model [3]. Further, we observed that the expression of c-Myc was also significantly decreased in BME-fed tumor as compared to control animals (Figure 2, panel C). Together, these results suggested that BME treatment reduced tumor growth by inhibiting tumor cell proliferation and c-Myc expression. We further examined the induction of apoptosis in control and BME-fed tumors. TUNEL positive cells were significantly higher in tumor from BME-fed mice as compared to control group (Figure 2, panel D).

### Bitter melon modulates Treg populations in the tumor microenvironment

**Figure 3:**
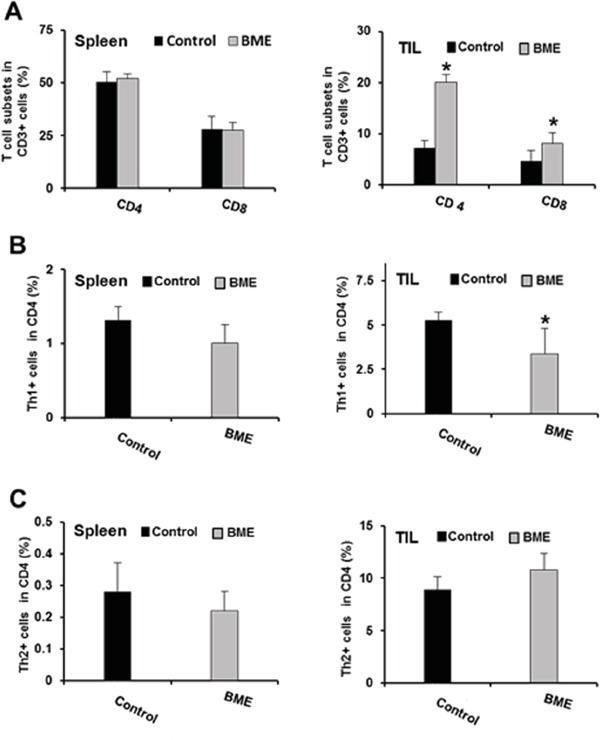
Effect of BME treatment on T cell immunophenotyping **A.** Expression of the CD4^+^ and CD8^+^ cells in spleens and tumors in both control and BME-fed groups. **B.** Expression of Th1 cell population in spleens and tumor of both control and BME-fed groups. **C.** Expression of Th2 cell population in spleens and tumor of both control and BME-fed groups. (*, p<0.05).

**Figure 4:**
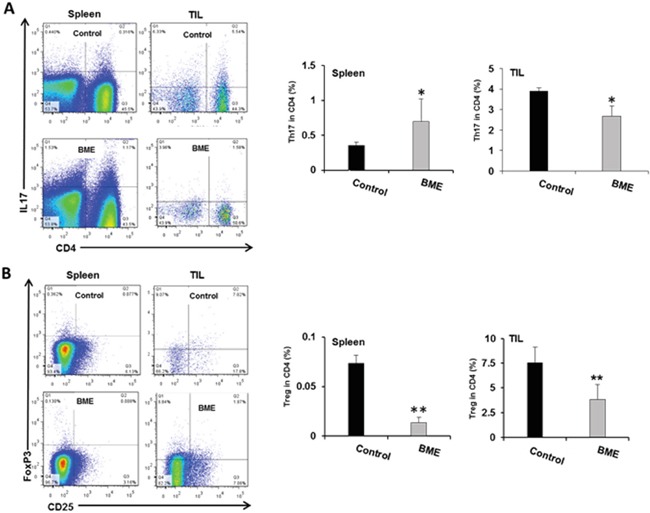
Modulation of tumor-infiltrating CD4+ T cell subsets in the mouse HNSCC model following BME feeding **A.** Representative dot plot distribution of Th17 (CD4^+^IL17^+^) cell population and quantitation of Th17 cell population from spleen and tumor respectively. **B.** Representative dot plot distribution of Treg (CD4^+^CD25^+^) cell population and quantitation of Treg cell population in spleens and tumors of both control and BME-fed groups (*, p<0.05).

Tumor microenvironment plays a critical role in defining the efficacy of chemotherapeutic drug as well as natural products in different cancer models [11]. Modulating the immune system to treat cancer is a major goal of immunotherapy [4]. Several immunomodulators including natural products have been investigated to modulate immune system activity to enhance the immune response against cancer [9, 11]. We therefore investigated whether BME treatment could also modulate the T cell numbers and their components in tumor bearing mice in this head and neck cancer model. We observed increased number of infiltrating T lymphocytes in the tumors of BME-fed group as compared to untreated group of mice (data not shown). We next studied the proportion of CD8^+^ and CD4^+^ cell populations in CD3^+^ lymphocyte. We observed that the splenic CD4^+^ and CD8^+^ population did not alter significantly following BME treatment; however both CD4^+^ and CD8^+^ subpopulations were significantly increased in tumors following BME feeding (Figure 3, panel A). We then analyzed the expression profile of CD4 subtypes, commonly modulated in tumor. Th1 and Th2 cells, two most common CD4^+^ subtypes, are generally modulated in tumors. Tumors can reduce Th1 cell population, which can secrete IFNγ, regulating the development and persistence of cytotoxic T cells. CD8^+^ T cells also secrete IFNγ which are known to be essential for tumor eradication. Interestingly, we did not observe a modification of the Th1 cell population in spleen, however, they were lower in BME-fed tumors (Figure 3, panel B). In addition, Th2 cell populations were not significantly modulated following BME treatment in spleens or tumors (Figure 3, panel C). Th17 cells, another subset of CD4^+^ cells, play dynamic role in the tumor microenvironment [12]. However, the functional impact of Th17 cells to tumor immunity remains unclear because it exhibited both pro- and anti-tumor function in various types of cancer models [12]. To investigate the status of Th17 cells in our model, we examined the Th17 cell populations in tumors and spleens of mice. We observed that Th17 populations were increased in the spleen, whereas, decreased in the tumors of BME-fed mice as compared to those of control mice (Figure 4, panel A). Treg cells are responsible for suppressing anti-tumor immunity in the tumor microenvironment. Immunosuppressive microenvironments induced by Treg cells present a major barrier for successful anti-tumor immunity and tumor immunotherapy [13]. Among all types of CD4^+^ subsets, Treg cells are one of the key components and play a very crucial role in immunosuppression. To investigate the potential impact of BME on Treg cells in HNSCC model, we determined the status and correlation of Treg cells in tumor as well as in the spleen of BME-fed mice. Our results demonstrated that the Treg (CD4^+^CD25^+^FoxP3^+^) cell population was significantly decreased in the spleen and also in the tumor of the BME-fed mice as compared to control animals (Figure 4, panel B), suggesting the immunomodulatory role of BME.

## DISCUSSION

In this study, we observed that BME feeding in HNSCC syngeneic mouse model display reduced tumor growth without any apparent sign of toxicity. The identification of molecular targets is important in terms of monitoring the clinical efficacy of cancer therapeutic strategies. PCNA plays a crucial role as an integral component of the eukaryotic DNA replication machinery in normal cellular growth and differentiation [14], and is required for cell growth and cell cycle progression in mammalian cells. c-Myc, a proto-oncogene, plays an important role in cancer cell survival, growth and apoptosis [15]. c-Myc is known to be overexpressed in various cancers including HNSCC. Our data strongly demonstrated a reduced expression of PCNA and c-Myc in tumors of BME-fed animals in HNSCC syngeneic mouse model. We also observed induction of apoptosis in BME-fed mice as compared to control mice.

Naturally occurring anti-inflammatory or immunomodulatory plant extracts contribute to an anticancer effect by modulation of immune signaling pathways. In this present study, we observed that infiltrating T lymphocytes were increased after BME treatment. The CD3^+^ lymphocyte population was significantly increased in tumors after BME feeding. However, CD4^+^IFNγ^+^ cell population or CD8^+^IFNγ^+^ CTL cell population were not altered in tumors or splenocytes in our model after BME feeding.

Tumor-infiltrating Treg cells demonstrate immunosuppressive microenvironment, inhibit effective anti-tumor immunity and act as a major hurdle for the better treatment of cancer [7]. Treg cells secret anti-inflammatory cytokines such as IL-10 and facilitate in escaping of immunosurveillance in the growing tumor [16]. The recruitment of Treg cells is more common in tumor bearing mice and the depletion of these cells potentiates antitumor activity [17]. The frequency of Treg cells is increased at tumor sites and among the peripheral blood lymphocytes of patients with HNSCC [6]. Our study suggested that BME feeding consistently reduces the recruitment of CD4^+^CD25^+^ Foxp3 expression in tumors and spleens. Foxp3 is a well characterized marker of Treg cells and its expression has been implicated a requite for Treg cell function [17].

CD4^+^ Th17 cells play a dynamic role in tumor immunity though there are opposing reports regarding its function [12]. Th17 level has been shown to be increased in patients with ovarian cancer, cervical cancer, hypopharyngeal carcinoma, gastric cancer, and lung cancer, with a shift from Th17 to Treg cells in advanced disease [18]. In contrast, the premalignant epidermal squamous lesions in mouse model demonstrated increased induction of Th17 cells to coincide with tumor regression [19]. Therefore, the role of Th17 cells in cancer development has been conflicting and may depend on the tumor microenvironment milieu. Increase number of Th17 cells in the tumor microenvironment is associated with the progression of several cancers including head and neck cancer [20, 21]. Our study indicated that Th17 cell populations were decreased in the tumors, but not in spleens following BME feeding.

Taken together, our results strongly support that BME acts at different levels in controlling the tumor growth. BME feeding controls cell growth by directly modulating cell proliferation. We also examined the effect of BME in tumor microenvironment and observed immunomodulatory activity. BME exerts its effect in reducing Treg cell populations, although the underlying mechanism remains to be elucidated. Therefore, these findings offer a new insight into how bitter melon extract inhibits head and neck tumor growth by modulating cell proliferation and Treg populations, with implications for how to control tumor-infiltrating lymphocytes and tumor progression. To our knowledge, this is the first report describing the immunomodulatory role of bitter melon in suppression of HNSCC tumor growth.

## MATERIALS AND METHODS

### Cell line and BME preparation

Mouse head and neck squamous cell carcinoma (HNSCC) cell line SCCVII was kind gift from Dr. J. Martin Brown at Stanford University, and maintained in Waymouth's medium containing 15% fetal bovine serum (FBS) and 1% of penicillin/streptomycin at 37°C in 5% CO_2_ humidified incubator. BME was prepared from the Chinese variety of young bitter melons (raw and green) as discussed previously [3, 22]. Briefly, BME was extracted using a household juicer and centrifuged at 560 x g at 4°C for 30 min, freeze dried at -45°C for 72 h and stored at −80°C until used for feeding studies. We prepared a stock of 0.15 g/ml in water, aliquoted, and used for *in vitro* cell culture work and 100 μl/mouse for oral gavage.

### *In vivo* studies

Animal experiments were performed according to the NIH guidelines, following a protocol approved by the Institutional Animal Care and Use Committee (IACUC) of Saint Louis University. C3H/HeNTac mice (6 weeks old females) were purchased from Taconic Farms Inc. (USA) and housed in a specific pathogen free facility. SCCVII (5×10^4^) cells were suspended in 100 μl serum free medium and mixed with 40% BD-Matrigel (BD Bioscience) and implanted subcutaneously into the flank of mouse. Mice were randomized into two groups (n=5), group one received 100 μl of water (control group) and the other group received 100 μl of BME by oral gavage (experimental group) 5 days/week. Tumor volume was measured every 3 days using a digital caliper. Tumor volume was calculated according to the formula L × W^2^ × 0.5 (L = length; W = width; all parameters in millimeters). When tumor volume reached ~1500 mm^3^, all mice were sacrificed. For immunohistochemical and histopathological studies, some portion of tumors were fixed into 10% neutral buffered formalin and embedded into paraffin and some part was snap-frozen and stored at −80°C for Western blot analysis immediately after excision. Remaining part of tumors were collected in media and processed for flow cytometry analysis. Intact spleen was also collected from each mouse and processed for isolation of splenocytes followed by flow cytometry. This animal experiment was repeated four times.

### Immunohistochemical studies

Immunohistochemical analysis of PCNA was performed using antibody against PCNA as described previously [10].

### Western blot analysis

Snap frozen tumor samples were lysed in 2× SDS sample buffer and subjected to Western blot analysis using specific antibodies for expression of proliferating cell nuclear antigen (PCNA) (Santa Cruz Biotechnology) and c-Myc (Cell Signaling). The blots were reprobed with GAPDH and actin antibody to compare protein load in each lane.

### TUNEL assay

Measurement of apoptosis in the tumors was examined by TUNEL assay using the TdT-Fragel DNA Fragmentation detection Kit (BioRad), following manufacturer's protocol. TUNEL positive cells were quantitated. Slides were scanned on an Olympus VS120 (Olympus Scientific Solutions Americas, Waltham, MA) dedicated slide scanner at 20x objective magnification. DAPI and DAPI+FITC signals in nuclei were quantitated using CellSens Dimension (Olympus Scientific Solutions Americas, Waltham, MA).

### Isolation of splenocytes and tumor infiltrating lymphocytes (TIL)

Spleen from each mouse was harvested and splenic cell suspensions were prepared by mincing the tissues using a wire mesh. Cell suspension was passed through a 40 μm cell strainer. The lysis of RBC was performed using red blood cell lysis buffer (Sigma). Subsequently, splenocytes were washed three times using phosphate buffer saline (PBS, pH7.4) and then re-suspended in FACS staining buffer (PBS containing 0.5% BSA) and processed for flow cytometry.

Tumor cells were also harvested by mincing the tissue through a wire mess from each tumor. Cells were passed through the 40 μm strainer and the single cell suspension was layered gently over the ficoll in the ratio of 1:1. After centrifugation, resulting cells were collected from the interface. The cells were then washed with PBS and re-suspended in RPMI1640 media containing 10% FBS and processed for flow cytometry.

### Analysis of immune cell by flow cytometry

T cells and their subsets were analyzed by differential expression of surface markers as well as by their intracellular cytokine profile. Briefly, cells were stained with different surface markers, such as anti-mouse CD3e (clone BM10-37, BD Biosciences), anti-mouse CD4 (clone RM4-5, BD Biosciences), anti-mouse CD8a (clone53-6.7, BD Biosciences) and anti-mouse CD25 (clone PC61, BD Biosciences). For analysis of the expression of intra cellular cytokines, cells were stimulated with 50 ng of PMA and 1 μg of ionomycin for 4 h in the presence of Golgi stop (BD Biosciences) to inhibit the secretion of the cytokines from the cells. Cells were then washed with staining buffer, fixed and permealized with BD cytofix and cytoperm^TM^Plus (BD Biosciences) along with 0.2% Tween 20 for 20 minutes. Next, cells were stained with anti-mouse IL4 (clone 11B11, BD Biosciences), anti-mouse IFN-γ (clone XMG1.2, BD Biosciences), anti-mouse IL-17A (clone TC11-18H10) and anti-mouse Foxp3 (clone MF23, BD Biosciences). Brilliant stain buffer (BD Biosciences) and ultra comp beads (eBioscience, San Diego) were used for the dilution of the stains and compensation respectively. All the stained cells were analyzed by LSRII flow cytometer (BD Biosciences) and the data was evaluated by FlowJo software.

### Statistical analysis

Two-tailed Student's t-test was used for statistical analysis. All statistical analysis was performed using Statistical Analysis System software (Graphpad Prism 6) and p values <0.05 were considered significant.

## SUPPLEMENTARY FIGURE



## Abbreviations

BME: bitter melon extract
HNSCC: head and neck squamous cell carcinoma
TIL: tumor infiltrating lymphocytes
