# Supplementary material for: Immunomodulatory role of bitter melon extract in inhibition of head and neck squamous cell carcinoma growth
Source: Oncotarget. 2016 Apr 21;7(22):33202–9. doi: 10.18632/oncotarget.8898 (PMC5078086; doi:10.18632/oncotarget.8898)
Supplement: Supplementary file 1 [file oncotarget-07-33202-s001.pdf]

SUPPLEMENTARY FIGURE

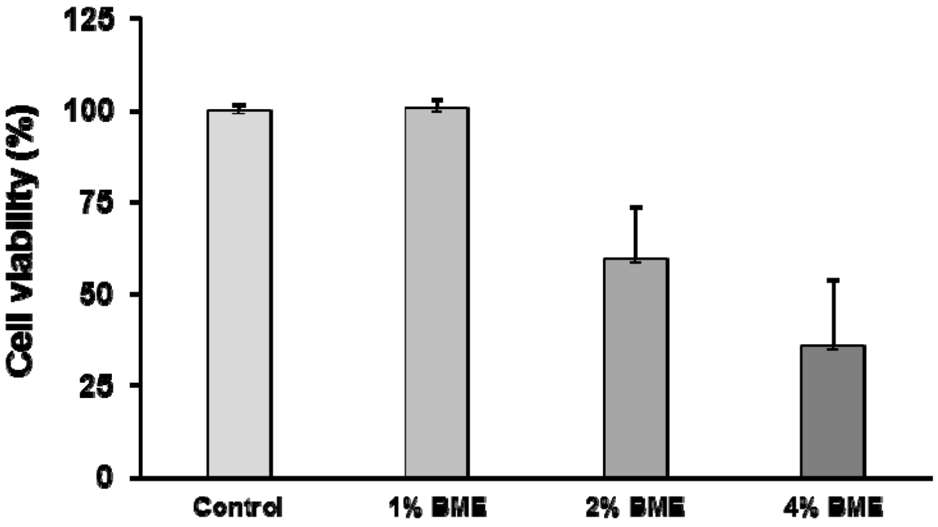

Supplementary Figure S1: BME inhibits HNSCC cell proliferation. HNSCC cells (SCCVII) were treated with indicated concentrations of BME and cell viability was measured after 72 h.
